# The evaluation of an automated mariPOC SARS‐CoV‐2 antigen test compared to RT‐qPCR SARS‐CoV‐2 assay and comparison of its sensitivity in Delta‐ and Omicron‐variant samples

**DOI:** 10.1111/irv.13048

**Published:** 2022-09-06

**Authors:** Marcela Krutova, Marie Brajerova, Zdenek Kepka, Ales Briksi, Petr Hubacek, Pavel Drevinek

**Affiliations:** ^1^ Department of Medical Microbiology, 2nd Faculty of Medicine Charles University and Motol University Hospital Prague Czech Republic

**Keywords:** antigen testing, Delta, Omicron, SARS‐CoV‐2, sensitivity

## Abstract

**Background:**

The rapid diagnostics tests for SARS‐CoV‐2 antigen vary in their sensitivities, and moreover, genomic mutations may further affect the performance of the assays. We aimed to evaluate the analytical performance of an automated antigen assay and compare its sensitivity in Delta‐ and Omicron‐variant positive clinical samples.

**Material and methods:**

The analytical performance of an automated mariPOC SARS‐CoV‐2 antigen test was evaluated on a population of community‐dwelling subjects with mild respiratory symptoms or being asymptomatic investigated by the RT‐qPCR Allplex™ SARS‐CoV‐2 assay. The sensitivity and specificity of the antigen test were evaluated on prospective 621 nasopharyngeal swabs along with oropharyngeal swabs. The sensitivity regarding variants determined by the Allplex™ SARS‐CoV‐2 Variant assays was analysed in additional, retrospective 158 Delta and 59 Omicron samples.

**Results:**

The overall sensitivity of the antigen test in prospective samples was 77.9% (113/145; 95% confidence interval [CI] 70.3–84.4) with the specificity of 99.8% (95% CI 98.8–100). Regarding the variant, the sensitivity was higher in Omicron‐variant samples, 93.2% (55/59; 95% CI 83.5–98.1), compared to Delta‐variant samples, 71.5% (113/158; 95% CI 63.8–78.4; p = .001).

**Conclusion:**

In community‐dwelling subjects with mild respiratory symptoms or being asymptomatic, the automated mariPOC SARS‐CoV‐2 antigen test showed high sensitivity over 98.0% in subgroup samples with cycle threshold (Ct) values < 25. Regarding the variant, the antigen test sensitivity was higher in the Omicron‐variant samples compared to the Delta‐variant samples. The analytical performance of the antigen test can differ between the SARS‐CoV‐2 variants, and a re‐evaluation should be performed for new circulating lineages.

## 1 INTRODUCTION

Since the first cluster of SARS‐CoV‐2 (severe acute respiratory syndrome coronavirus) cases in Wuhan (China) in December 2019 was reported,^1^, ^2^ the spread of this virus has been responsible for continuing to increase the number of Covid‐19 infections and attributed deaths.^3^

The introduction of robust testing has to be one of the key tools in preventing and controlling the spread of the virus. Although RT‐qPCR is the gold standard for SARS‐CoV‐2 detection, rapid antigen tests have been widely used due to quick results and limited access to PCR detection. The range of commercial assays for antigen SARS‐CoV‐2 detection has grown exponentially with the testing requirements, but the diagnostic sensitivity of individual tests has shown to be very variable in clinical use contrasting sensitivities stated by the manufacturers.^4^, ^5^ To standardize antigen testing in European Union, the requirements for the Covid‐19 rapid antigen test performance evaluation were formulated and a list of rapid antigen tests that are considered appropriate for use was released and regularly updated.^6^

However, the sensitivity of the detection (in both molecular and antigen tests) can be further affected by genetic changes in the SARS‐CoV‐2 genome due to an intrinsically error‐prone RNA polymerase, which it employs for replication.^7^ These genetic changes confer a competitive advantage, for example, increasing transmissibility enabling rapid spread and the predominance of certain virus variants. Until now, five variants of concern (VOCs) have been declared by the World Health Organization.^8^

To date, the latest VOC, Omicron (also known as B.1.1.529), was identified on 5 November 2021 in South Africa and its occurrence has been reported worldwide. Compared to other circulating variants, the Omicron genome contains an excessive number of mutations, mainly in the *Spike* gene. However, the four mutations have been also found in Nucleocapsid (N) protein (P13L, GERS30G, R203K and G204R),^9^ the target protein of rapid antigen tests,^5^ suggesting that diagnostic performance of already validated assays should be reassessed also on SARS‐CoV‐2 Omicron‐variant samples. The need is supported by the study of Bekliz *et al.* where variable sensitivity for Omicron antigen detection compared to earlier circulating SARS‐CoV‐2 lineages and the other VOCs was observed; when three assays had comparable sensitivity, but in four antigen tests, significantly lower sensitivity (p < .001) was observed.^10^

This study aimed to evaluate the performance of an automated mariPOC SARS‐CoV‐2 antigen assay and compare its sensitivity in SARS‐CoV‐2 Delta‐ and Omicron‐variant clinical samples.

## 2 MATERIAL AND METHODS

### 2.1 Study settings

The samples for the study were taken at the collection site at Motol University Hospital, Prague, Czech Republic.^11^ The sampled individuals included symptomatic outpatients suspected for Covid‐19, asymptomatic or symptomatic contacts of Covid‐19 laboratory‐confirmed cases, students and/or employees with positive SARS‐CoV‐2 antigen tests from preventive testing at school or work and asymptomatic citizens who required testing for the purposes of their Covid Pass.

Nasopharyngeal swabs along with oropharyngeal swabs were sampled in accordance with the international specimen collection guidelines^12^ and placed in 2 ml of virus stabilization tube VACUETTE® (Greiner Bio‐One Preanalytics, Austria) containing a phosphate‐buffered saline solution at a pH of 7.4 ± 0.2 (VTM).

### 2.2 The index SARS‐CoV‐2 antigen test

The index antigen test in the study was an automated mariPOC SARS‐CoV‐2 test (ArcDia International Ltd, Finland) that targets a conserved epitope in the N‐protein.^13^ The volume of 650 μl of VTM was diluted with 650 μl of test sample buffer, vortexed and inserted into the instrument. In the prospective part of the study, antigen test results were unknown by those interpreting RT‐qPCR results.

The sensitivity and specificity of the antigen test were evaluated in a prospective part of the study that was carried out between 8 November and 15 November 2021. A volume of 650 μl of VTM was used for the antigen testing immediately after sample collection. The residual VTM was stored temporarily at 4°C, and 200 μl of VTM was later used for nucleic acid extraction and further analyses.

The sensitivity of the antigen test in relation to the SARS‐CoV‐2 Delta or Omicron variant was carried out from the samples collected in six sampling days in October and November 2021 (for the Delta variant) and four sampling days in January 2022 (for the Omicron variant). The residual VTM of RT‐qPCR SARS‐CoV‐2‐positive samples irrespectively to RT‐qPCR cycle threshold (Ct) value determined as Delta variant (n = 158) or Omicron variant (n = 59) was analysed within 24–48 h after sampling. The residual VTM samples were stored at 4°C upon analysis.

### 2.3 SARS‐CoV‐2 RT‐qPCR detection and virus‐variant determination

The RNA extraction was performed with Viral Nucleic Acid Extraction kit (Zybio, Chongqing, China) on the EXM3000 instrument (Zybio) and analysed with RT‐qPCR Allplex™ SARS‐CoV‐2 assay (Seegene, Seoul, Republic of Korea), run on the CFX96 PCR cycler (Bio‐Rad, Hercules, CA, USA), targeting N, E and RdRP/S genes; the nucleocapsid gene target was used as a reference.

For the detection of SARS‐CoV‐2 VOCs and variants of interest (VOIs), the RT‐qPCR SARS‐CoV‐2‐positive samples were investigated by the Allplex™ SARS‐CoV‐2 Variants I and II Assays (Seegene). The Allplex™ SARS‐CoV‐2 Variant I kit detects E484K, HV69/70 deletion and N501Y in the S gene, and the Variant II detects K417N, K417T, L452R and W152C in the S gene. SARS‐CoV‐2 Delta‐variant (B.1.617.2) positive sample was determined by the presence of L452R substitutions and the SARS‐CoV‐2 Omicron variant (B.1.1.529) was determined by the presence of N501Y and K417N substitutions and HV69/70 deletion. In addition, the Sanger sequencing of the S gene in randomly selected samples was performed to monitor current SARS‐CoV‐2 epidemiology. The combination of mutations was unique to Omicron and Delta variants during the time period of the study.

### 2.4 Statistics

Data were collected in Excel 2019 (Microsoft, Redmond WA, USA) (supporting information). Statistical calculations were conducted using the R statistical software Version 3.5.1^14^ and/or using IBM SPSS Statistics. The Clopper–Pearson exact method was used to calculate confidence intervals (CIs). The Mann–Whitney U test, the Cohen's kappa coefficient and the chi‐square test were used for statistical significance evaluation. The significance level was set to .05.

## 3 RESULTS

### 3.1 The study population

For the evaluation of sensitivity and specificity of the antigen test in the prospective samples, a total of 621 samples were analysed. The median age of the tested individuals was 30 years (interquartile range [IQR] 12–48, ranged between 39 days and 78 years); 60.1% of those tested were females (n = 373) (supporting information).

For evaluation of the sensitivity of the antigen test regarding variants in the retrospective samples, a total of 217 SARS‐CoV‐2 PCR‐positive samples were analysed; 158 were determined as Delta variant and 59 as Omicron variant by the commercial discriminatory PCR assay. In Delta‐variant samples, the median age of the tested individuals was 28.5 years (IQR 14–47, ranged between 1 and 94 years); 53.8% (n = 85) of those tested were females. In Omicron samples, the age of tested persons ranged between 1 and 80 years (median 28 years, IQR 18–38); 56.9% (n = 33) of samples were from females (supporting information).

### 3.2 The sensitivity and specificity of the antigen test on prospective samples compared to RT‐qPCR

From 621 samples analysed, 145 were SARS‐CoV‐2 PCR positive (23.4%) based on the RT‐qPCR. Among these, 113/145 samples resulted positive and 475/476 resulted negative to both antigen and RT‐qPCR detection, showing 94.7% inter‐assay concordance, with a substantial agreement based on the Cohen's kappa coefficient (κ = 0.8396; 95% CI = 0.787–0.892).

**FIGURE 1:**
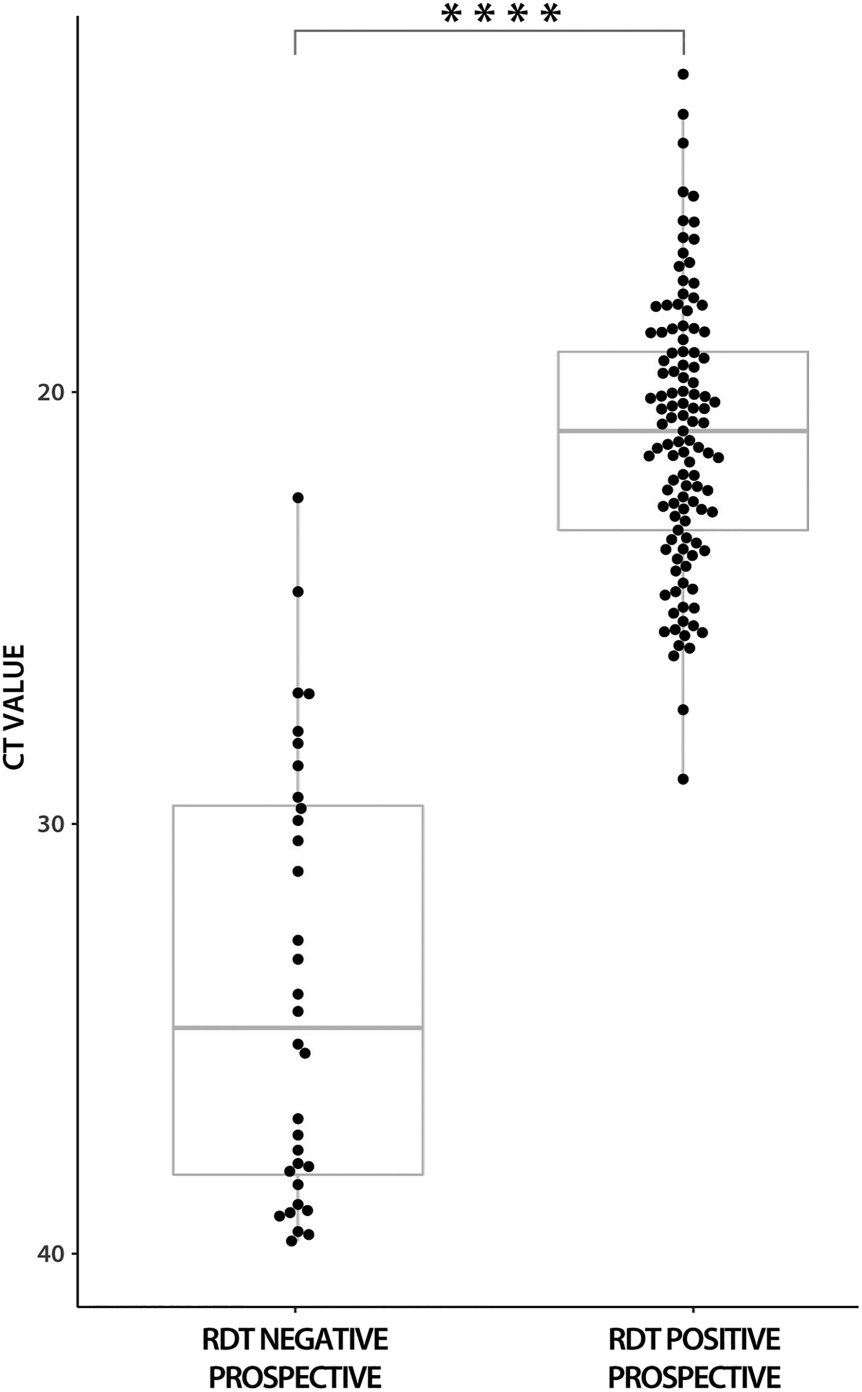
Prospective antigen diagnostic test results in comparison to RT‐qPCR Ct values. Ct, cycle threshold; RDT, rapid diagnostic test. ****p = .0001 (Mann–Whitney U test)

Considering RT‐qPCR as a reference, the overall sensitivity of the antigen test in prospective samples was 77.9% (95% CI 70.3–84.4) and the specificity was 99.8% (95% CI 98.8–100). The Ct values were significantly lower in antigen test‐positive samples compared to antigen test‐negative samples (mean 21.0; SD 3.08 vs. mean 33.74; SD 5.06; p = .0001; Mann–Whitney U test) (Figure 1).

When grouped according to Ct values, the sensitivity of the antigen test results was 98.0% (95% CI 93.0–99.8) for Ct values < 25, 91.9% (95% CI 85.6–96.0) for Ct values < 30 and 89.7% (95% CI 83.0–94.4) for Ct values < 33 (Table 1).

**TABLE 1:** Sensitivity (positive percentage agreement) of the mariPOC SARS‐CoV‐2 test when compared with the qRT‐PCR results

RT‐qPCR Ct‐value groups	PCR positive	RDT positive	Sensitivity agreement (%)	95% CI
Prospective study				
Ct < 25	101	99	98.0	93.0–99.8
Ct < 30	123	113	91.9	85.6–96.0
Ct < 33	126	113	89.7	83.0–94.4
Overall positivity	145	113	77.9	70.3–84.4
Delta variant				
Ct < 25	71	70	98.6	92.4–100
Ct < 30	121	111	91.7	85.3–96.0
Ct < 33	135	112	83.0	75.6–88.9
Overall positivity	158	113	71.5	63.8–78.4
Omicron variant				
Ct < 25	36	36	100	90.3–100
Ct < 30	53	51	96.2	87.0–99.5
Ct < 33	58	55	94.8	85.6–98.9
Overall positivity	59	55	93.2	83.5–98.1

Abbreviations: CI, confidence interval; Ct, cycle threshold; RDT, rapid diagnostic test.

### 3.3 The sensitivity of the antigen test in Delta‐ and Omicron‐variant samples

**FIGURE 2:**
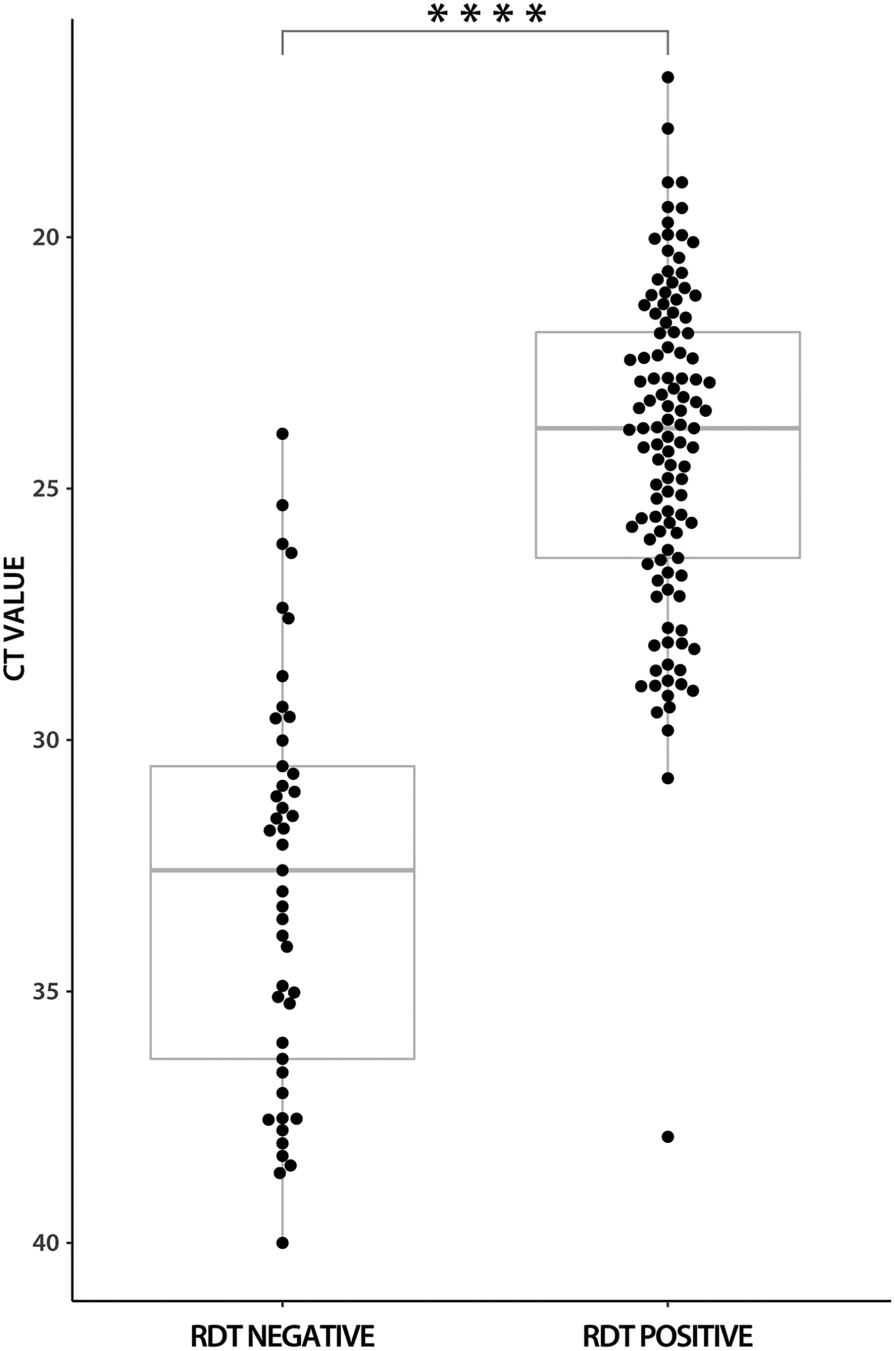
Antigen diagnostic test results in comparison to RT‐qPCR Ct values in Delta‐variant samples. Ct, cycle threshold; RDT, rapid diagnostic test. ****p = .0001 (Mann–Whitney U test)

In Delta‐variant samples, the antigen test was positive in 113/158 samples, reaching a sensitivity of 71.5% (95% CI 63.8–78.4). The Ct values were significantly lower in antigen test‐positive samples compared to antigen test‐negative samples (mean 24.21; SD 3.3 vs. mean 32.86; SD 4.02; p = .0001; Mann–Whitney U test) (Figure 2). When grouped according to Ct values, the sensitivities were as follows: 98.6% (95% CI 92.4–100) for samples with Ct values < 25, 91.7% (95% CI 85.3–96.0) for samples with Ct values < 30 and 83.0% (95% CI 75.6–88.9) for samples with Ct values < 33 (Table 1).

**FIGURE 3:**
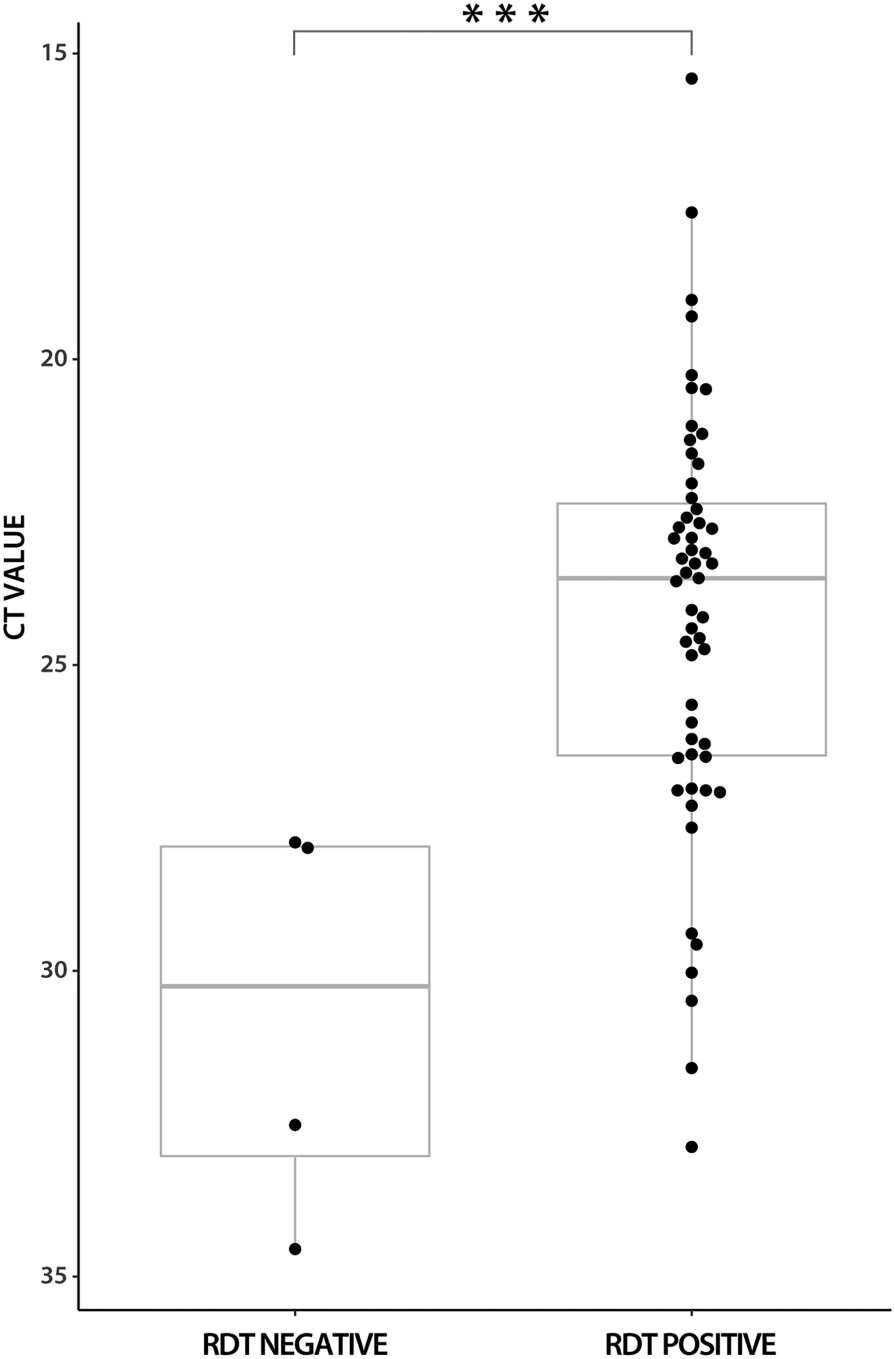
Antigen diagnostic test results in comparison to RT‐qPCR Ct values in Omicron‐variant samples. Ct, cycle threshold; RDT, rapid diagnostic test. ***p = .001 (Mann–Whitney U test)

In Omicron‐variant samples, the antigen test was positive in 55/59; the sensitivity was 93.2% (95% CI 83.5–98.1). The Ct values were significantly lower in the antigen test‐positive samples compared to the antigen test‐negative samples (mean 24.22; SD 3.41 vs. mean 30.74; SD 3.33; p = .001; Mann–Whitney U test) (Figure 3). For samples with Ct values < 25, the antigen test showed sensitivity of 100% (95% CI 90.3–100.0), for Ct values < 30, it was 96.2% (95% CI 87.0–99.5) and for Ct values < 33, it was 94.8% (95% CI 85.6–98.9) (Table 1).

**FIGURE 4:**
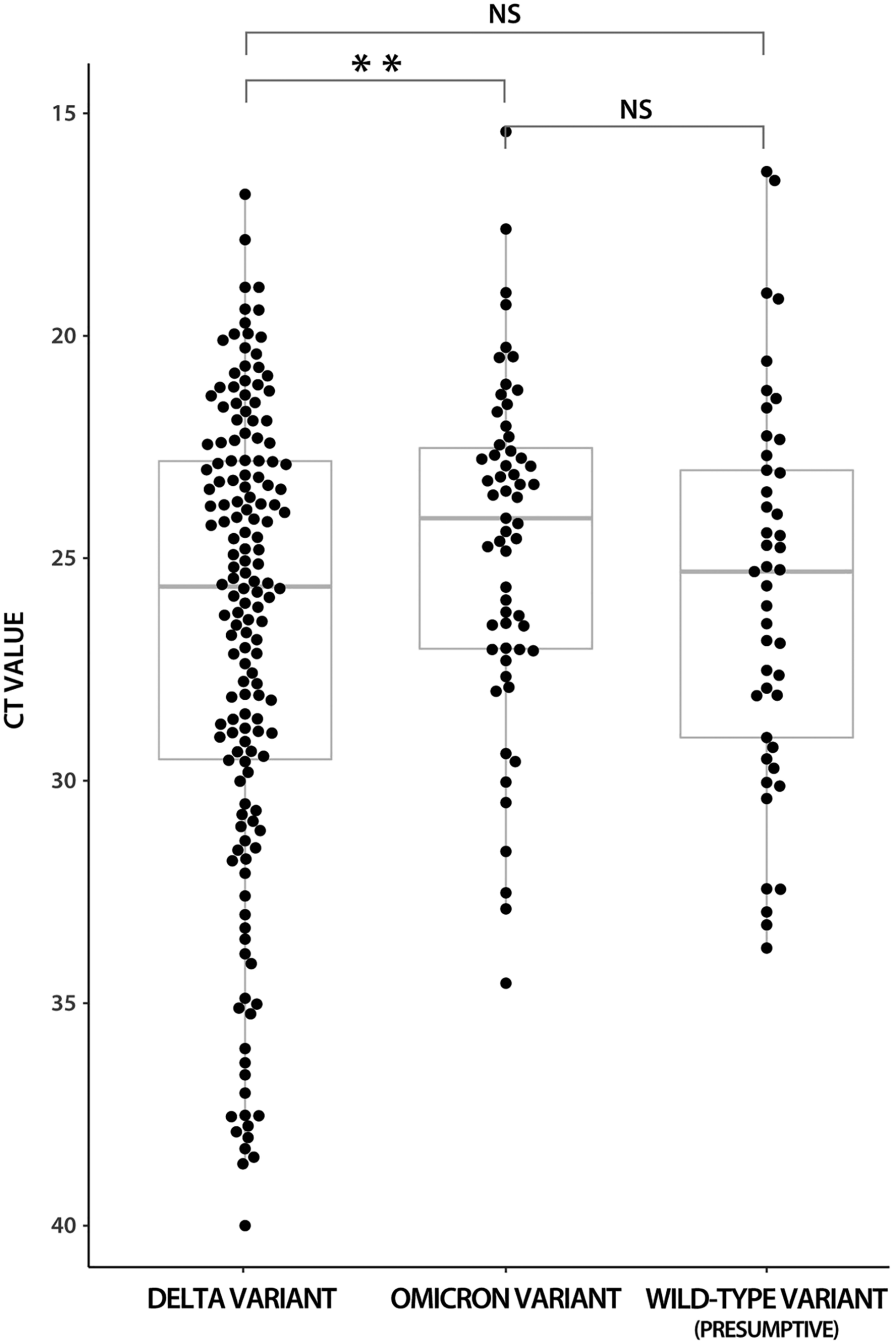
RT‐qPCR Ct values distribution in wild‐type (presumptive),^13^ Delta‐ and Omicron‐variant samples (this study). Ct, cycle threshold; NS, non‐significant (Mann–Whitney U test). **p = .028

The overall sensitivity of the antigen test in Omicron samples was significantly higher compared to the Delta‐variant samples (93.2% vs. 71.5%, p = .001; chi‐square test); however, when the distribution of Ct values between Omicron and Delta samples was compared, the Ct values of Omicron‐variant samples were slightly lower (mean 24.66; SD 3.76 vs. mean 26.67; SD 5.24; p = .028; Mann–Whitney U test) (Figure 4).

## 4 DISCUSSION

Although the RT‐qPCR is the method recommended for confirmation of Covid‐19 diagnosis, the use of rapid antigen tests has also gained widespread acceptance as an alternative method due to low cost, short turnaround time and easy interpretation of the results. The sensitivities of commercial antigen tests vary, and importantly, the mutations in the SARS‐CoV‐2 genome can have a potential impact on the diagnostic test performance.^5^, ^10^ Thus, we performed this study with the aim of evaluating the performance of an automated antigen assay in prospective samples and comparing its sensitivity regarding the Delta and Omicron variants.

The mariPOC SARS‐CoV‐2 antigen test is an automated assay where nucleocapsid proteins are detected based on sandwich immunoassay and two‐photon excitation fluorescent measurements of individual microparticles by confocal microscopy.^13^ Through the different principles of the assay, compared to the lateral flow test based on the double‐antibody sandwich principle,^15^ the automated immunoassay with signal amplification and sensitive detection is expected to be more sensitive.

In the prospective samples, the overall sensitivity of the antigen test was 77.9% (95% CI 70.3–84.4), which exceeded the average sensitivity of tests reporting both sensitivity and specificity reviewed in the Cochrane database (68.9%, 95% CI 61.8–75.1).^5^ The higher sensitivity was also achieved in a subgroup of samples with Ct values < 25 (98.0%, 95% CI 93.0–99.8 vs. 94.5, 95% CI 91.0–96.7) and in samples with Ct values < 33 (89.7%, 95% CI 83.0–94.4 vs. 82.5, 95% CI 74.0–88.6).^5^ It should be noted that the recommended sample in the mariPOC test is a native nasopharyngeal swab specimen suspended into 1.3 ml of the sample buffer; thus, the dilution of the sample in VTM in our study could possibly lower the assay sensitivity.

The correlation of viability of the SARS‐CoV‐2 virus and Ct value was investigated previously.^16^, ^17^ In the study of La Scola *et al.*, samples with Ct values of 13–17 all led to a positive culture, but in samples with Ct of 33, culture positivity decreased to 12% and no culture was obtained from samples with Ct > 34. The authors concluded that patients with Ct above 33–34 are not contagious.^16^ In contrast to the above‐mentioned study, Bullard *et al.* observed SARS‐CoV‐2 infectivity in Vero cells for RT‐PCR Ct < 24.^17^

When considering the above‐mentioned infectivity thresholds, only one case would be left under‐detected in the subgroup of samples of Ct < 24. When calculated antigen test sensitivity for individuals with Ct values ≤ 34, 16 antigen‐negative but PCR‐positive samples would not be included and the overall sensitivity of antigen test would increase from 77.9% to 89.0%.

The clinical sensitivity of an automated mariPOC SARS‐CoV‐2 antigen test has been evaluated previously in 58 frozen qRT‐PCR‐positive nasopharyngeal samples from two specimen cohorts.^13^ In the first cohort of 13 patients, the swabs were suspended directly into the mariPOC sample buffer or first into saline (range 0.1–0.65 ml) and the sensitivity was 100% (13/13; 95% CI 75.3–100.0), but Ct values of qRT‐PCR were available only for four samples (ranging between 21 and 28). In the second cohort of 45 specimens with qRT‐PCR Ct values from 16 to 34, the overall sensitivity was 84.4% (38/45; 95% CI 70.5–93.5),^13^ which is comparable to prospective samples' sensitivity result of 77.9% (95% CI 70.3–84.4; p = .46) from our study.

Considering the SARS‐CoV‐2 variant, the sensitivity of the antigen test was significantly higher, reaching 93.2% in the Omicron‐variant samples compared to the Delta‐variant samples (71.5%; p = .001). Unfortunately, the data on variants of the SARS‐CoV‐2 were not available for samples from the study of Koskinen *et al*.^13^ We can only assume that patients were infected with wild‐type strains of SARS‐CoV‐2 because, until 18 December 2020, sequencing‐based surveillance conducted in the Hospital District of Helsinki and Uusimaa, Finland, reported only wild‐type strains of SARS‐CoV‐2^18^ and samples of the second cohort (with Ct values available) from the specimen library of the Finnish Institute of Health and Welfare, Helsinki, Finland, were received in April 2020 (personal communication with J. M. Koskinen). When compared with the sensitivities of the antigen tests in presumptive wild‐type samples, the sensitivity was similar to the Omicron‐ or Delta‐variant samples (p > .05; chi‐square test) with no difference in Ct values distribution (mean 25.75; SD 4.29 vs. mean 26.59; SD 5.15 vs. mean 24.66; SD 3.76) (Figure 4).

Although Ct values in Omicron‐variant samples were slightly lower (mean 24.66; SD 3.76 vs. mean 26.67; SD 5.24; p = .028), the difference in sensitivities among antigen assays may be caused by structural changes in the N‐protein^9^ that may affect the interaction with antibodies, as was observed in Spike protein.^19^ As shown in Figures 2 and 3, more positive index antigen test measurements were present in Omicron‐variant samples with Ct values above 30 compared to Delta‐variant samples. The role of different viral loads regarding the SARS‐CoV‐2 variant is less likely because no significant differences in viral loads were observed when compared with wild‐type and Delta‐variant samples or Delta‐ and Omicron‐variant samples. Even lower viral loads of patients infected with the Omicron variant than those of the Delta‐variant infected patients were reported.^20^, ^21^, ^22^, ^23^

## 5 CONCLUSION

In community‐dwelling subjects with mild respiratory symptoms or being asymptomatic, the automated mariPOC SARS‐CoV‐2 antigen test showed high sensitivity of 98.0% (95% CI 93.0–99.8) in subgroup samples with Ct values < 25. Regarding the variant, the test sensitivity was higher in the Omicron‐variant samples compared to the Delta‐variant samples, 93.2% (55/59; 95% CI 83.8–97.3) vs. 71.5% (113/158, 95% CI 64.0–78.0; p = .001). The analytical performance of antigen tests can differ between SARS‐CoV‐2 variants; thus, a re‐evaluation should be performed for new dominant variants.

## CONFLICT OF INTEREST

No conflict of interest declared.

## ETHICS

The ethics committee of the Motol University Hospital approved the assay comparison based on anonymized samples (EK‐1260/21) and waived for informed consent because data and samples were collected as part of the clinical routine.

## AUTHOR CONTRIBUTIONS

**Marcela Krutova**: Conceptualization; formal analysis; writing – original draft. **Marie Brajerova**: Formal analysis (supporting); investigation (supporting); software (lead); visualization (lead); writing – review and editing. **Zdenek Kepka**: Formal analysis (supporting); investigation (supporting); writing – review and editing. **Ales Briksi**: Investigation (supporting); formal analysis; conceptualization; writing – review and editing. **Petr Hubacek**: Investigation (supporting); conceptualization; writing – review and editing. **Pavel Drevinek**: Conceptualization (supporting); supervision (lead); writing – review and editing.

## Supporting information


**Data S1.** Supporting Information

## Data Availability

Available anonymized patient information and measurement data are available in the supporting information.
